# Use of the Pessary in the Prevention of Preterm Delivery

**DOI:** 10.1055/s-0038-1676511

**Published:** 2019-01

**Authors:** Thayane Delazari Corrêa, Ester Gomes Amorim, Jade Aimée Guimarães Tomazelli, Mário Dias Corrêa

**Affiliations:** 1 Departament of Obstetrics and Gynecology, Universidade Federal de Minas Gerais, Belo Horizonte, MG, Brazil

**Keywords:** pessary, preterm birth, delivery, premature, short cervix, pessário, nascimento prematuro, parto, prematuridade, colo curto

## Abstract

**Objective** The gestational complication most associated with perinatal mortality and morbidity is spontaneous preterm birth with gestational age < 37 weeks. Therefore, it is necessary to identify its risk factors and attempt its prevention. The benefits of the pessary in prematurity are under investigation. Our objective was to analyze the use of the pessary in the prevention of preterm births in published studies, and to compare its efficacy with other methods.

**Methods** Randomized clinical trials published between 2010 and 2018 were selected from electronic databases. Studies on multiple gestations were excluded.

**Results** Two studies were in favor of the pessary as a preventive method, one study was contrary to the method and another two showed no statistically significant difference. The meta-analysis showed no statistical difference with the use of a cervical pessary in the reduction of births < 37 (odds ratio [OR]: 0.63; confidence interval [95% CI]: 0.38–1.06) and < 34 weeks (OR: 0.74; 95% CI: 0.35–1.57)

**Conclusion** The pooled data available to date seems to show a lack of efficacy of the cervical pessary in the prevention of preterm birth, although the heterogeneity of the studies made comparisons more difficult.

## Abstract (pt)

**Resumo:**

**Objetivo** O parto com idade gestacional < 37 semanas é a complicação gestacional mais associada à mortalidade e morbidade perinatal, sendo necessária a identificação de seus fatores de risco e a tentativa de sua prevenção. Os benefícios do pessário na prematuridade estão sendo investigados. Nosso objetivo foi analisar os estudos publicados sobre uso do pessário na prevenção do parto pretermo e comparar sua eficácia perante outros métodos.

**Métodos** Foram selecionados estudos clínicos randomizados publicados entre 2010 e 2018, extraídos de bases eletrônicas de dados. Estudos de gestações múltiplas foram excluídos.

**Resultados** Dois estudos se mostraram a favor do pessário como método preventivo, um estudo foi contrário ao método, e outros dois não demonstraram diferença estatisticamente significativa. A meta-análise não mostrou diferença significativa no uso do pessário na redução de nascimentos < 37 (razão de chance [RC]: 0,63; intervalo de confiança [IC 95%]: 0,38–1,06) e < 34 semanas (RC: 0,74; IC 95%: 0,35–1,57).

**Conclusão** Os dados agrupados disponíveis até o momento parecem mostrar uma falta de eficácia do pessário cervical na prevenção do parto pretermo, embora a heterogeneidade dos estudos tenha dificultado as comparações.

## Introduction

Nearly 15 million preterm births occur annually worldwide. Delivery at a gestational age < 37 weeks is the gestational complication more closely associated with perinatal mortality and morbidity.^1^ Children born prematurely still have a high risk of complications and hospital readmissions throughout life.^2^ ^3^

The etiology of preterm birth is multifactorial, and the history of preterm delivery is the most significant risk factor. Another important factor is the presence of a short cervix (< 25 mm) identified by transvaginal ultrasonography between 20 and 24 weeks of gestation.^2^ ^4^

A large reduction in mortality rates and in neonatal morbidity resulting from preterm deliveries will only be achieved with greater accuracy after the proper identification of women with risk factors for this complication and the development of efficient prevention strategy.^2^

One of the prevention strategies considered is the use of progesterone. Progesterone acts reducing the contractions of the uterine smooth muscle and decreasing the inflammatory process involved in the onset of labor. Progesterone is considered a key hormone for pregnancy maintenance, and if a decline of progesterone action occurs in the midtrimester, cervical shortening may occur, which would predispose the patient to preterm delivery. A blockade of progesterone action can lead to the clinical, biochemical and morphologic changes associated with cervical ripening.^5^ Progesterone has been shown to be effective in reducing the preterm delivery and neonatal mortality rates when compared with placebo.^3^ ^4^

Cervical cerclage is a surgical procedure first introduced by Shirodkar and McDonald in the mid-1950s, currently used prophylactically for women with second-trimester repetitive loss suggestive of cervical insufficiency. A history of previous preterm birth and of cervical changes in the ongoing gestation indicated by ultrasonography (cervical length < 25 mm before 24 weeks) and altered physical examination (cervical dilatation perceived in the physical examination before 24 weeks) are also recognized indications in the literature. Cervical cerclage consists of a suture of the uterine cervix, performed preferably at the beginning of the gestation (8–14 weeks), which acts as a physical barrier, as well as a biochemical one, by protecting the membranes from ascending pathogens.^2^ ^6^

An alternative approach could be the pessary, which is a device that has been used for the past 50 years.^7^ The pessary is a conical ring of silicone that is introduced inside the vagina until it encircles the entire cervix, closing the cervical canal and preventing its dilatation or shortening.^1^ ^8^ ^9^ It promotes a change in the cervical angle, reducing the direct pressure of the uterine contents in the canal, and may be a safer alternative to surgical cerclage because it is easily removable and does not require anesthesia.^3^ ^7^ ^9^ This device can be used from the diagnosis of a short cervix, usually around between 18 and 22 weeks of gestation, and is withdrawn by the obstetrician at between 36 and 37 weeks of gestation, at which age the fetus has better clinical and physiological conditions for survival.^7^

**Fig. 1:**
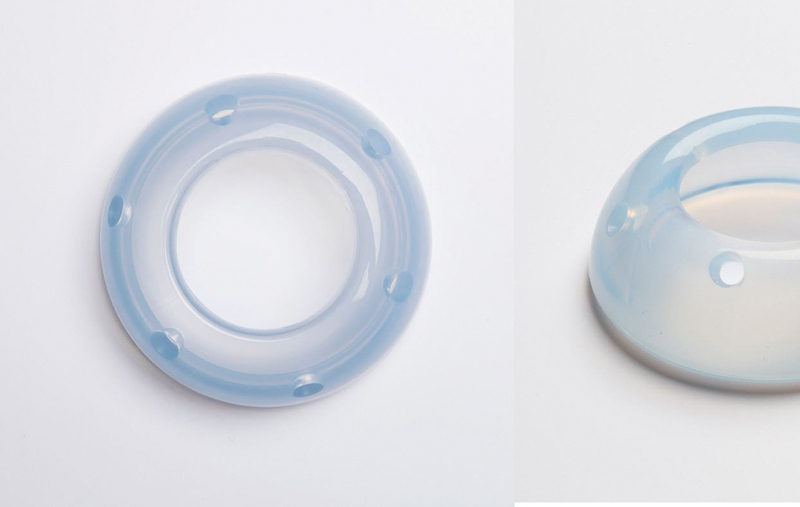
Picture of the ARABIN Cerclage Pessar Perforiert pessary.^10^

**Fig. 2:**
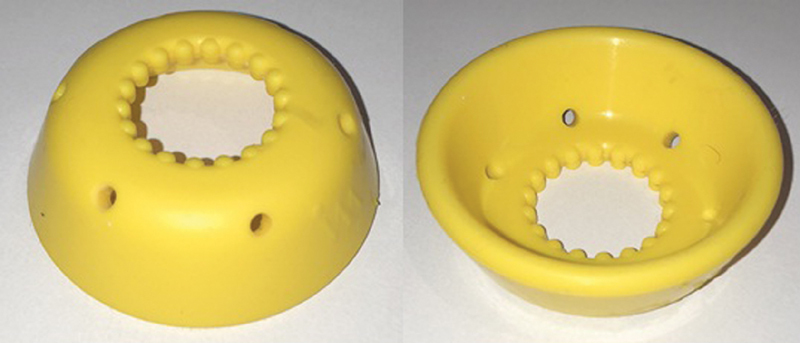
Picture of the PESSÁRIO AM INGÁMED^11^.

The ARABIN Cerclage Pessar Perforiert pessary (Dr. Arabin GmbH & Co., Witten, Germany)^10^ has three different diameters to better suit the uterine cervix. It has been released for use in Brazil about two years ago and we have an authorized representative in Brazil (Fig. 1). The AM Ingámed pessary (Ingámed, Maringá, PR, Brazil)^11^ is developed by a Brazilian company, made of silicone in order to better adapt to the cervix (Fig. 2). We do not have studies comparing the differences between the two types. In Brazil, the P5 study is underway to compare vaginal progesterone versus pessary and progesterone in patients with short cervix diagnosed by ultrasound, using the Ingámed pessary. The use of a cervical pessary in conjunction with intravaginal progesterone is shown to be a safe and feasible method for the prevention of preterm birth in women with a short midtrimester cervix. Moreover, this combined treatment has led to a pregnancy prolongation of ∼ 13.5 weeks, according to recent studies.^12^

Since it is a less invasive preventive method than cerclage, not dependent on hormonal supplementation, the pessary is assuming an important role in the medical practice among obstetricians.^2^ The present article aims to review the latest advances in the efficacy of this method in the management of patients at risk of preterm birth.

## Methods

Trials were identified by searching the literature in the PubMed, Scielo, EMBASE and Cochrane databases, between 2010 and 2018. The keywords used were *pessário* and *pessário* *cervical* and their correspondents in English, *pessary* and *cervical* *pessary*. The inclusion criteria were: articles with randomized controlled trials randomized clinical trials (RCTs) that evaluated the use of the pessary in the prevention of preterm births. Twenty-eight articles were found, and the ones that did not meet the criteria of the present study were excluded. The exclusion criteria were: articles published outside the period described, and those referring to the use of pessaries in multiple gestations.

The final review was based on 5 articles from RCTs analyzing the efficacy of the pessary in preventing preterm birth in single pregnancies, in which the expected primary ≥ 34 and ≥ 37 weeks.

**Fig. 3:**
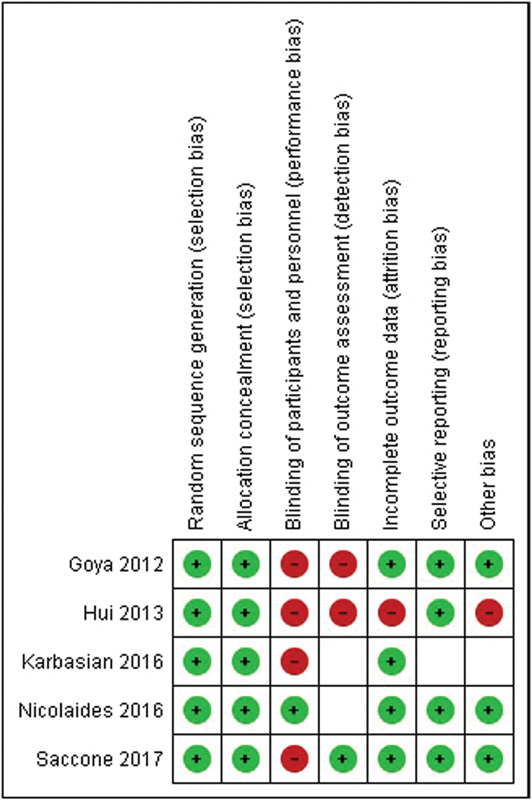
Summary of the risk of bias for each trial: minus sign: high risk of bias; plus sign: low risk of bias; blank space: unclear risk of bias.

The assessment of the quality of the included studies and of their risk of bias was performed according to the criteria outlined in the Cochrane Handbook for Systematic Reviews of Intervention.^13^ The results are shown on Fig. 3.

Two types of comparisons were made among these publications:

1) Vaginal progesterone versus pessary associated with vaginal progesterone.
2) Pessary versus expectant management.

The data analysis was completed independently by two authors (Corrêa Júnior, M. D., and Corrêa T. D.), using the Review Manager (RevMan), Version 5 software (Cochrane Collaboration, Copenhagen, Denmark). The summary measures were reported as relative risk (RR) with a 95% confidence interval (CI).

## Results

**Fig. 4:**
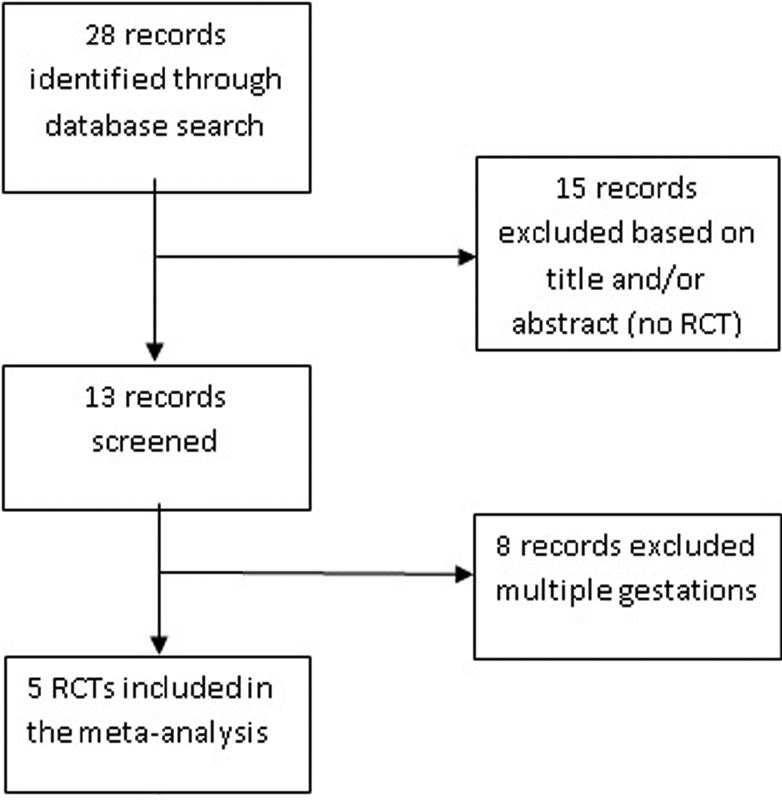
Flow diagram of identified studies.

Fig. 4 shows the flow diagram of the information through the different phases of the review. Thirteen studies were screened; eight trials including multiple gestations were excluded. Five RCTs were therefore included in the meta-analysis.

Blinding was considered not methodologically feasible, given the type of intervention, and none of the studies included was double-blinded. All of the five studies used the ARABIN Cerclage Pessar Perforiert.^10^

Two studies, Saccone et al (2017)^1^ and Karbasian et al (2016)^4^ compared the association of the pessary with vaginal progesterone with the use of progesterone alone. Although the studies have similar outlines, their results were different. Saccone et al (2017)^1^ observed a twofold lower preterm delivery rate with pessary use than in the progesterone-only group (7.3% for pessary associated with progesterone, and 15% for progesterone).

Karbasian et al (2016)^4^ did not observe a statistically significant difference in the preterm delivery rates between the two groups (19.7% for pessary associated with progesterone, and 16.4% for progesterone alone).

Studies comparing the use of pessary with expectant management also showed different outcomes. Nicolaides et al (2016)^3^ did not observe statistically significant differences when comparing the use of the pessary with expectant management (12% of premature parturition with pessary use, and 10.8% without any intervention).

Hui et al (2013)^9^ observed a 4.1% higher rate in the outcome of births before 34 weeks with the use of the pessary (9.4% in the pessary group, and 5.5% in the control group).

On the other hand, Goya et al (2012)^2^ observed in their study a 4.5-fold lower rate of preterm delivery with pessary use versus expectant behavior (6% and 27% of births before 34 weeks, respectively).

It is important to note that, in all of the studies, there was no significant difference between maternal or infant perinatal morbidity and mortality rates as a function of the choice of prevention method.

Vaginal discharge as the main side effect was found in four studies. Goya et al (2012)^2^ observed 100% of vaginal discharge in the cervical pessary group, and 46% in the expectant management group. Hui et al (2013)^9^ observed 47% of vaginal discharge in the cervical pessary group, and 21.8% in the control group. Nicolaides et al (2016)^3^ observed vaginal discharge in 46.8% of the pessary group versus 13.8% of the control group, and a high vaginal swab was obtained for bacteriologic examination; if the results showed infection, appropriate antibiotic therapy was administered. Saccone et al (2017)^1^ found vaginal discharge as a side effect in 8.7% of the pessary group, and in 46% of the control group. Karbasian et al (2016)^4^ do not describe vaginal discharge as a side effect. Table 1 summarizes the main findings of the analyzed studies.

**Table 1:** Main results of the analyzed studies

Author	N	Prematurity rate		RR	*P*-value
		Pessary	Control		
Goya et al (2012)^2^	380	6%	27%	0.18 (0.08–0.37)	< 0.0001
Hui et al (2013)^9^	108	9.4%	5.5%	1.04 (0.94–1.12)	0.46
Karbasian et al (2016)^4^	144	19.7%	16.4%	1.20 (0.60–2.41)	0.60
Nicolaides et al (2016)^3^	924	12%	10.8%	1.12 (0.75–1.69)	0.57
Saccone et al (2017)^1^	300	7.3%	15.3%	0.48 (0.24–0.95)	0.04

Abbreviation: RR, relative risk.

**Fig. 5:**
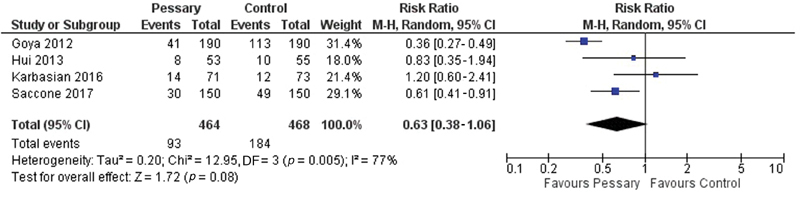
Meta-analysis of included studies for delivery < 37 weeks.

**Fig. 6:**
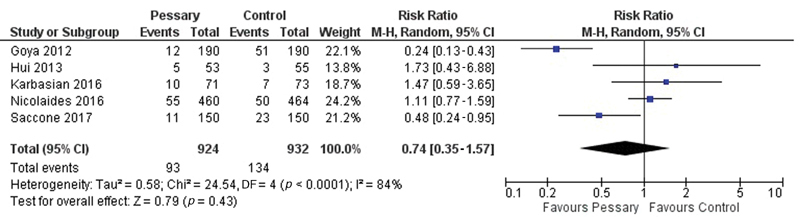
Meta-analysis of included studies for delivery < 34 weeks.

The comparison between these studies is difficult due to the methodologies used: pessary versus absence of intervention or vaginal progesterone. This difficulty is even greater when we consider that progesterone was also used in the group randomized to the use of the pessary in some studies, at the discretion of the attending physician. With this limitation in mind, we have performed a meta-analysis of the included studies, as shown in Figs. 5 and 6.

The meta-analysis showed no statistically significant difference in the use of the pessary with or without the association of vaginal progesterone compared with the group that did not use the pessary when the birth is < 37 weeks (RR: 0.63; 95% CI: 0.38–1.06), or < 34 weeks (RR: 0.74; 95% CI: 0.35–1.57).

## Discussion

Goya et al (2012)^2^ and Saccone et al (2017)^1^ obtained different results, concluding that the pessary could prevent preterm birth in a population of at-risk women, suitably selected by cervical screening on transvaginal ultrasonography performed during prenatal care obtained similar results. However, in the study by Goya et al, only 11% of the population was at high risk of spontaneous preterm birth due to previous history.

The divergence between the studies by Nicolaides et al (2016)^3^ and by Saccone et al (2017)^1^ raise the question of whether cervical pessaries can only be effective at a very low cervix length cutoff, since the mean cervical length in the study by Saccone et al (2017),^1^ which demonstrated a favorable outcome to the use of the pessary, was ∼ 12 mm, whereas in the study by Nicolaides et al (2016),^3^ the mean cervical length was ∼ 20 mm. In addition, the use of progesterone in the study by Nicolaides et al (2016)^3^ in pregnant women with cervical length ≤ 15 mm may have attenuated the benefit of the pessary in this group.

There were more women with previous preterm deliveries (16.5%) in the study by Nicolaides et al (2016),^3^ compared with the study by Goya et al (2012)^2^ (10.8%), which raises the question of whether the pessary can be effective only in women with a short cervix, but without a prior preterm delivery history. However, this issue could not be further analyzed due to the the fact that none of these trials reported prior spontaneous preterm birth or no prior spontaneous preterm birth as subgroups.

Saccone et al (2017)^1^ performed the only trial involving women with asymptomatic singleton pregnancies without prior spontaneous preterm birth, but with short cervical length detected by transvaginal ultrasound. The other four studies analyzed the same sample of patients with and without a history of prematurity, which may have influenced their outcome.

The differences presented between the analyzed studies show that a better evaluation is necessary before we generalize the favorable outcome of the pessary in the reduction of preterm delivery, considering, for example, low-risk women or different ethnic groups in the analysis.

For example, a more in-depth assessment is needed to clarify whether the study by Goya et al (2012)^2^ recruited women with additional risk factors that could be responsible for such a high baseline preterm rate (27%) compared with the study by Hui et al (2013)^9^ (9.4%). Perhaps the differences in the basal characteristics of the participating women could clarify this question.

Similarly to our analysis, a systematic review and meta-analysis by Saccone et al (2017)^14^ found that the ARABIN Pessary^10^ does not reduce the rate of spontaneous preterm delivery or improve the perinatal outcome, despite not having included the two studies included our analysis improve the perinatal outcome.

The cervical pessary was not associated with any harmful effects but was associated with a higher rate of vaginal discharge. Although significantly more patients in the pessary group commonly reported side effects, especially an increase in vaginal discharge, the rates of cervicovaginal infection did not differ significantly between the groups in the study by Nicolaides et al (2016),^3^ which was the only trial that used a high vaginal swab for bacteriological examination.

According to Alfirevic et al (2013),^15^ cerclage, vaginal progesterone, and the pessary appear to have similar effectiveness as management strategies in women with a singleton pregnancy, previous spontaneous preterm birth, and a short cervix. However, Norman et al (2016)^16^ performed the largest randomized trial of vaginal progesterone for the prevention of preterm birth in high-risk women, and found no difference between vaginal progesterone and placebo, concluding that the efficacy of progesterone in improving outcomes is either non-existent or weak. However, this study included a very heterogeneous group of patients and was underpowered to detect a meaningful difference. In another study performed by Hassan et al (2011),^17^ it was found that the administration of vaginal progesterone to women with a short cervix was associated with a reduction in the rate of preterm delivery < 33 weeks, < 35 weeks, and < 28 weeks of gestation. Although there is a small number of publications on the use of the pessary, with a diversity of results, indicating the need for more research, the use of this non-hormonal, accessible, and less invasive method for the pregnant woman, which is easily removable when necessary, has been gaining space in the medical practice.^1^ ^18^

## Conclusion

From the analyzed studies, we can conclude that the cervical pessary seems to show a lack of efficacy in the prevention of preterm birth. However, it is not possible to determine its inferiority in the reduction of preterm births when compared with other methods due to the heterogeneity of the existing studies. Its association with progesterone also requires a better evaluation.

## References

[1] Saccone G Maruotti G M Giudicepietro A Martinelli P; Italian Preterm Birth Prevention (IPP) Working Group. Effect of cervical pessary on spontaneous preterm birth in women with singleton pregnancies and short cervical length: a randomized clinical trial JAMA 2017 318 23 2317 2324. Doi: 10.1001/jama.2017.18956 29260226 10.1001/jama.2017.18956 PMC5820698

[2] Goya M Pratcorona L Merced C, et al; Pesario Cervical para Evitar Prematuridad (PECEP) Trial Group. Cervical pessary in pregnant women with a short cervix (PECEP): an open-label randomised controlled trial Lancet 2012 379(9828):1800 1806. Doi: 10.1016/S0140-6736(12)60030-0 22475493 10.1016/S0140-6736(12)60030-0

[3] Nicolaides K H Syngelaki A Poon L C, et al. A randomized trial of a cervical pessary to prevent preterm singleton birth N Engl J Med 2016 374 11 1044 1052. Doi: 10.1056/NEJMoa1511014 26981934 10.1056/NEJMoa1511014

[4] Karbasian N Sheikh M Pirjani R Hazrati S Tara F Hantoushzadeh S Combined treatment with cervical pessary and vaginal progesterone for the prevention of preterm birth: A randomized clinical trial J Obstet Gynaecol Res 2016 42 12 1673 1679. Doi: 10.1111/jog.13138 27718280 10.1111/jog.13138

[5] Cruz-Melguizo S San-Frutos L Martínez-Payo C, et al. Cervical pessary compared with vaginal progesterone for preventing early preterm birth: a randomized controlled trial Obstet Gynecol 2018 132 04 907 915. Doi: 10.1097/AOG.0000000000002884 30204689 10.1097/AOG.0000000000002884

[6] Suhag A Berghella V Cervical cerclage Clin Obstet Gynecol 2014 57 03 557 567. Doi: 10.1097/GRF.0000000000000044 24979354 10.1097/GRF.0000000000000044

[7] Cross R G Treatment of habitual abortion due to cervical incompetence Lancet 1959 274 127. Doi: 10.1016/S0140-6736(59)92242-1

[8] Abdel-Aleem H Shaaban O M Abdel-Aleem M A Cervical pessary for preventing preterm birth Cochrane Database Syst Rev 2013 05 CD007873. Doi: 10.1002/14651858.CD007873.pub3 23728668 10.1002/14651858.CD007873.pub3 PMC6491132

[9] Hui S YA Chor C M Lau T K Lao T T Leung T Y Cerclage pessary for preventing preterm birth in women with a singleton pregnancy and a short cervix at 20 to 24 weeks: a randomized controlled trial Am J Perinatol 2013 30 04 283 288. Doi: 10.1055/s-0032-1322550 22875662 10.1055/s-0032-1322550

[10] *ARABIN® Cerclage Pessar perforiert.* https://dr-arabin.de/produkt/arabin-cerclage-pessar-perforiert/. Accessed September 25, 2018

[11] *Pessário Parto Prematuro*. 2015. http://www.ingamed.com.br/produtos-detalhe/51/pessario-parto-prematuro. Accessed September 25, 2018

[12] Daskalakis G Zacharakis D Theodora M, et al. Safety and efficacy of the cervical pessary combined with vaginal progesterone for the prevention of spontaneous preterm birth J Perinat Med 2018 46 05 531 537. Doi: 10.1515/jpm-2017-0009 29055173 10.1515/jpm-2017-0009

[13] Higgins J PT Green S *Cochrane Handbook for Systematic Reviews of Interventions Version 5.10*. The Cochrane Colaboration; 2011 http://handbook-5-1.cochrane.org/. Accessed September 15, 2018

[14] Saccone G Ciardulli A Xodo S, et al. Cervical pessary for preventing preterm birth in singleton pregnancies with short cervical length: a systematic review and meta-analysis J Ultrasound Med 2017 36 08 1535 1543. Doi: 10.7863/ultra.16.08054 28398701 10.7863/ultra.16.08054

[15] Alfirevic Z Owen J Carreras Moratonas E Sharp A N Szychowski J M Goya M Vaginal progesterone, cerclage or cervical pessary for preventing preterm birth in asymptomatic singleton pregnant women with a history of preterm birth and a sonographic short cervix Ultrasound Obstet Gynecol 2013 41 02 146 151. Doi: 10.1002/uog.12300 22991337 10.1002/uog.12300

[16] Norman J E Marlow N Messow C M, et al; OPPTIMUM study group. Vaginal progesterone prophylaxis for preterm birth (the OPPTIMUM study): a multicentre, randomised, double-blind trial Lancet 2016 387(10033):2106 2116. Doi: 10.1016/S0140-6736(16)00350-0 26921136 10.1016/S0140-6736(16)00350-0 PMC5406617

[17] Hassan S S Romero R Vidyadhari D, et al; PREGNANT Trial. Vaginal progesterone reduces the rate of preterm birth in women with a sonographic short cervix: a multicenter, randomized, double-blind, placebo-controlled trial Ultrasound Obstet Gynecol 2011 38 01 18 31. Doi: 10.1002/uog.9017 21472815 10.1002/uog.9017 PMC3482512

[18] Arabin B Halbesma J R Vork F Hübener M van Eyck J Is treatment with vaginal pessaries an option in patients with a sonographically detected short cervix? J Perinat Med 2003 31 02 122 133. Doi: 10.1515/JPM.2003.017 12747228 10.1515/JPM.2003.017

